# Midwifery abdication – is it acknowledged or discussed within the midwifery literature: An integrative review

**DOI:** 10.18332/ejm/92529

**Published:** 2018-06-27

**Authors:** Elaine Jefford, Julie Jomeen, Margie Wallin

**Affiliations:** 1 Southern Cross University, Australia; 2 University of Hull, Hull, UK

**Keywords:** decision-making, professionalism, accountability, midwifery practice, midwifery abdication, women-centred

## Abstract

**INTRODUCTION:**

In this review we explore the concept of Midwifery Abdication and whether it is acknowledged or discussed within the midwifery literature.

**METHODS:**

A modified Whittemore and Knafl integrative review framework of 2005 enabled consideration of quantitative and qualitative literature. A total of 1508 papers were located. Duplicate records were removed, leaving 1197 records. All titles, abstracts, or case facts were reviewed using a framework derived from the definition of *Midwifery Abdication*. Three qualitative studies were selected for analysis; the NICE Quality Appraisal Checklist was used to determine study quality.

**RESULTS:**

*Midwifery* Abdication occurs, as reported within the wider midwifery literature, and indicated in three studies from different countries. However, the original constructs need to be widened to include: ‘external perceptions of midwifery practice’ and ‘how can reflection facilitate change’. The extent of philosophy in these environments leads to the adoption of midwifery philosophy failure. Such an environment impacts on a midwife’s ability to fully exercise autonomy, and to advocate for normality and women. This renders *Midwifery Abdication* almost inevitable or at least very difficult to prevent. A midwife’s professional identity, environmental hierarchy and associated culture of social obedience, acceptance and finding one’s place, all act as influencing factors in abdication.

**CONCLUSIONS:**

Midwifery education needs to ensure that midwives are prepared and able to embrace their professional status as independent practitioners. Promotion of reflexive practice to facilitate personal and professional change is warranted. Practice policies that are not supportive of a midwife’s professional autonomy and scope of practice reinforce the technocratic work environment.

## INTRODUCTION

Midwifery is regulated by professional and regulatory bodies as well as the legal system. These bodies ensure that midwives are suitably educated and qualified to competently and ethically practice midwifery. These bodies in turn complement the legislative framework. Global standards and law may vary, nevertheless the ultimate aim is to ensure public safety. Consequently, the midwife must provide safe, effective, high quality and risk limited care, whilst honouring the woman’s right to make decisions about her own care^1-6^. To transverse such terrain may well place the midwife in the contested space between being accountable to the woman and her birth experience, on one hand, and being accountable to a variety of legal, professional and regulatory frameworks, on the other. The complexity of trying to successfully navigate this contested space may inadvertently result in a midwife abdicating her professional responsibilities^7^. Jefford first applied the term *Midwifery Abdication* in 2012 to such incidents^8^. It is defined as:

*‘. . . a midwife surrenders one’s voice and/or forsakes one’s midwifery skills and/or knowledge, consciously or unconsciously, failing to fulfil and be accountable for one’s own professional behaviour in accordance with professional frameworks as (primary) maternity care provider for the woman’*.

The aim of this review is to determine if the concept of *Midwifery Abdication* is acknowledged in other midwifery literature. The concept, whilst potentially contentious and somewhat unpalatable should not be disregarded. Explicit recognition of this construct facilitates the understanding of the challenging context within which midwives work. This review is not intended to be critical of midwives.

### Background

Jefford and Jomeen^7^ explored further the concept of *Midwifery Abdication*, which was first identified by Jefford^8^, and identified three influencing interrelated constructs: 1) internalized perceptions of midwifery practice, 2) knowing but failing to act and 3) prioritization of the woman’s needs.

### Internalized perceptions of midwifery practice

Based upon personal perceptions over a period of time, a midwife develops confidence in her/his own value as a ‘good’ midwife who rebuffs actions perceived as resulting in ‘poor’ midwifery care. Midwives cultivate attitudes and behaviours to support these positive feelings, thus protecting selfassurance and conviction in their interactions with women, peers and colleagues. This positive self-image, and associated self-esteem, is significant in obtaining and maintaining one’s own social identity within the midwifery profession. This subjective assessment may or may not be linked to one’s midwifery philosophy. It may or may not be linked with a need to create a beautiful birthing experience for the woman. Alternatively, it may or may not be linked with the midwife’s personal experience of being the recipient of good midwifery care. To be perceived as a ‘good’ midwife by those within and outside the birthing environment is therefore important to one’s sense of self-identity and sense of belonging^9^. If something or someone threatens these assumptions, it can result in behaviour that may result in a midwife abdicating her professional role.

### Knowing but failing to act

A midwife’s knowledge and scope of practice are embedded within regulatory, professional and legal accountability^1-6^. A midwife’s scope of practice encompasses understanding, supporting and optimising psychophysiology during pregnancy, labour and the post-natal period^10^. Further, a midwife takes responsibility for recognising any departure from the normal progression of pregnancy, labour and postnatal period and is required to consult and/or refer to other healthcare professionals^11^.

Some midwives, however, may abdicate their professional role despite knowing such boundaries exist, as a result of perceiving herself or himself to be disempowered by someone, or something within or outside the childbearing setting. Communication, cultural safety and/or entering an environment with raised stress levels were found to be contributing factors to *Midwifery Abdication*^7^.

England and Morgan^12^ believe effective communication is a learnt skill, which requires continuous conscious effort to actively improve interactions. Yet communication is complex, multifaceted and contextual, which can be influenced by diverse, single or multiple elements. Such is the case within a childbearing context. Examples of such elements include: a woman may be in an altered state of consciousness from which she should not be disturbed; health professionals’ gate keeping information versus providing information so the fundamental ethos that the woman is the final decision-maker of her own care is met; partner and/or family influence; birth being a rapidly changing situation; and medical dominance^7,8^. Integral to this, is the enactment of the midwife–woman relationship. Hunter^15^ identifies four types of exchanges that can impact upon the midwife–woman relationship and the care a midwife provides: balanced, rejected, reversed and unsustainable exchanges. Singly or collectively these elements can derail effective communication and ultimately result in a midwife feeling disempowered to act despite having the pre-requisite knowledge and skills, thus abdicating her professional midwifery role.

### Prioritization of the woman’s needs

A birth plan is an effective communication tool. It provides a woman with an opportunity to communicate her unique values, needs and wishes about her impending labour. Birth plans are predominately written and negotiated during the antenatal period. Increased birth satisfaction occurs when a woman’s requests are fulfilled^13^. Yet childbirth is unpredictable and deviations from normal may occur. In such cases, the midwife must inform the woman and if necessary renegotiate the birth plan^3,4,14^.

However, Jefford^8^ noted this was not always the case for midwives in an Australian study. A midwife may abdicate her professional role in a particular circumstance because she may believe and wish to ‘rescue’ the situation to achieve the woman’s ideal birth, as set out in the antenatal birth plan^7^. Significantly, however, in such circumstances, the woman does not know that the decisions she made in the antenatal period are no longer congruent with the unfolding clinical scenario. It is argued, therefore, that the woman becomes disempowered and potentially enters unknowingly into a vulnerable and unsafe place^7^.

*Midwifery Abdication* was derived empirically from a study that explored midwives from all models of midwifery/ maternity care within Australia^8^. These midwives gave accounts of caring for women in labour. However, it is important to explore whether the construct can be validated more widely in the literature and before any claims can be made about its utility for midwifery practice; an integrative literature review was therefore undertaken.

## METHODS

This review used a modified Whittemore and Knafl^16^ framework for synthesizing research studies consisting of four stages: problem identification, literature search, data analysis and presentation of findings. When synthesizing literature, three main ways in which studies are related occur: direct comparability, challenging points or when an amalgamated line of argument becomes visible^16^.

### Problem identification

Whittemore and Knafl^16^ suggest that the problem needs to be identified. We contend that the problem is the safety of the woman and her baby, and that the organisation and midwifery profession maybe at risk if a midwife abdicates her/ his professional role. The article by Jefford and Jomeen^7^ was used as the key paper, because it identifies and explains the concept of *Midwifery Abdication*. The purpose of this review is to explore if this concept is acknowledged or discussed within the midwifery literature, and if so, in what context.

### Literature search

Two of the authors performed the literature search within three databases — CINAHL (Cumulative Index to Nursing and Allied Health Literature), Medline and Scopus. The first two were selected for their discipline focus. Scopus was also searched — both for its high quality multidisciplinary coverage, and because it utilises Embase index terms and includes all Embase citations (direct access to Embase was not available to the researchers). As the MIDIRS Midwifery Digest is indexed within CINAHL, the MIDIRS database was not independently searched.

The initial search looked for the phrase *Midwifery Abdication, and retrieved a single paper: Midwifery Abdication: A Finding from an Interpretive Study*^7^.

As a result, the authors agreed to include synonymous terms, related to the concept of abdication including: accountability, consultation, empowerment, negligence, referral and responsibility. All terms were searched, either as formal subject headings or within the title or abstract, to increase relevance. The search was limited to scholarly English language papers, published between 2005 and 2017, as a 12-year span provides adequate representation of the current literature. Limits for quantitative and qualitative publication types were applied separately, to facilitate the evaluation process. No relevant quantitative studies were located, and the documented search strategy reflects the qualitative publication formats included.

**Figure 1:**
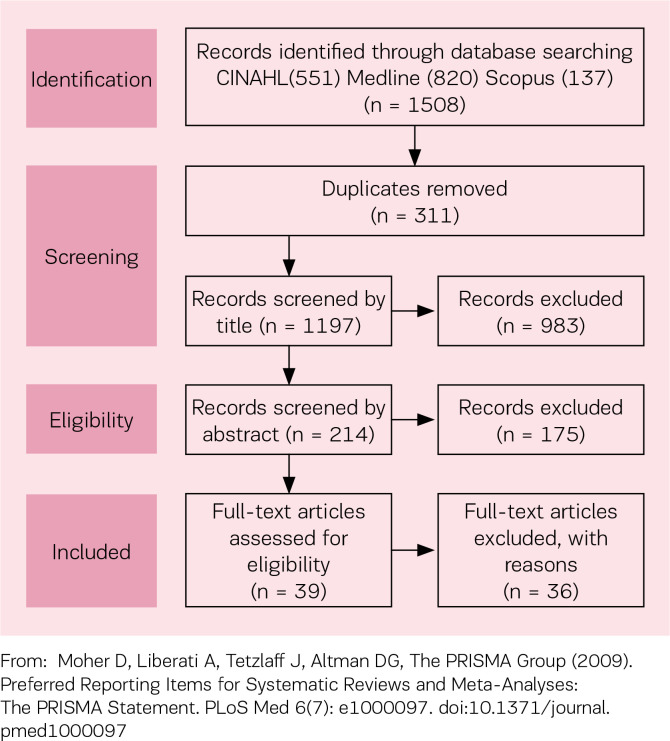
PRISMA 2009 Flow Diagram

Table 1 provides the detailed search strategy. A total of 1508 papers were located (CINAHL 551, Medline 820 and Scopus 137). Duplicate records (311) were removed, leaving 1197 records. All titles, abstracts or case facts were reviewed by two of the authors using the framework offered by the definition of *Midwifery Abdication* and the three identified influencing interrelated constructs ‘internalized perceptions of midwifery practice’, ‘knowing but failing to act’, and ‘prioritization of the woman’s needs’. Any paper that did not have at least one *Midwifery Abdication* construct, on reading the article title, abstract or case facts, was excluded. To enhance rigour and ensure validity, this part of the integrative review process was checked by the third author. All three authors independently read the remaining 39 papers analysing for content aligned with at least one construct. Each author presented her findings. Each author had rejected the same 36 papers for reasons such as title, abstract or case facts that were inconsistent with the content, and thus did not meet the inclusion criteria of the present review. Each author selected the same three articles for further analysis as their title, abstract or case facts and content were aligned and had at least one of the three identified influencing interrelated constructs within the definition of Midwifery Abdication (Figure 1)^17^. Analysis of reference lists occurred, with the aim of seeking any additional relevant articles^16^. No papers were added to those for review.

**Table 1:** Search strategies

*Database*	*Search*	*Limits*
**CINAHL**	((MH ‘Midwifery’) OR (MH ‘Students, Nurse Midwifery’) OR (MH ‘Students, Midwifery’) OR (MH ‘Education, Midwifery’) OR (MH ‘Research, Midwifery’) OR (MH ‘Nurse-Midwifery Service’) OR (MH ‘Education, Nurse Midwifery’) OR (MH ‘Royal College of Midwives’) OR (MH ‘Australian Nursing and Midwifery Council’) OR (MH ‘Nursing and Midwifery Council’) OR (MH ‘Nurse Midwifery’) OR (MH ‘Midwifery Service’) OR (MH ‘English National Board for Nursing, Midwifery and Health Visiting’)) AND ((MH ‘Accountability’) OR (MH ‘Competence (Legal)’) OR (MH ‘Liability, Legal’) OR (MH ‘Evidence, Legal’) OR (MH ‘Negligence’) OR (MH ‘Empowerment’) OR (MH ‘Powerlessness’) OR (MH ‘ Referral and Consultation’) OR TI ( abdicat* OR responsib* OR accountab* OR negligen* OR liab* OR empower* OR disempower* OR powerless* OR refer* OR consult*) OR AB ( abdicat* OR responsib* OR accountab* OR negligen* OR liab* OR empower* OR disempower* OR powerless* OR refer* OR consult*)	Limiters - Scholarly (Peer Reviewed) Journals; Published Date: 2005-2017; English Language; Publication Type: Abstract, Book, Book Chapter, Brief Item, Case Study, Doctoral Dissertation, Editorial, Interview, Journal Article, Legal Case, Masters Thesis, Meta Analysis, Meta Synthesis, Nurse Practice Acts, Practice Guidelines, Research, Review, Standards, Systematic Review
**Medline**	((MH ‘Midwifery’) OR (MH ‘Nurse Midwives’)) AND ((MH ‘Social Responsibility’) OR (MH ‘Liability, Legal’) OR (MH ‘Legal Cases’) OR (MH ‘Jurisprudence’) OR (MH ‘Malpractice’) OR (MH ‘Defensive Medicine’) OR (MH ‘Power (Psychology)’) OR (MH ‘Referral and Consultation’))OR AB ( abdicat* OR responsib* OR accountab* OR negligen* OR liab* OR empower* OR disempower* OR powerless* OR refer* OR consult*) OR TI ( abdicat* OR responsib* OR accountab* OR negligen* OR liab* OR empower* OR disempower* OR powerless* OR refer* OR consult*))	Limiters - Date of Publication: 2005-2017; English Language; Publication Type: Case Reports, Comment, Comparative Study, Editorial, Evaluation Studies, Government Publications, Guideline, Interview, Introductory Journal Article, Journal Article, Legal Cases, Legislation, Meta-Analysis, Multicenter Study, Practice Guideline, Review.
**Scopus**	(TITLE-ABS-KEY ( midwi*) AND TITLE-ABS-KEY (abdicat* OR responsib*) OR accountab* OR negligen* OR liab* OR empower* OR disempower* OR powerless* OR refer* OR consult* ))	Limited by date 2005-2017; English language

As part of the literature search, three legal cases were identified within the retrieved records. Following discussion by the authors, it was decided to exclude these cases from this integrative review as their focus was about coroner cases and professional hearings where practitioners were removed from the professional register. Further, a different approach to interpretation would have been required.

### Data evaluation

The National Institute for Health and Clinical Excellence (NICE) Quality Appraisal Checklist for Qualitative Studies^18^ was the critical appraisal tool used to guide data evaluation. Two of the authors independently read the three articles using the 14-point criteria of the NICE tool 18. Each of the 14-point criteria have sub-questions that guide the analysis in a systematic way (Table 2). Upon completion of the analysis the authors compared their findings and agreement was reached.

**Table 2:** Extract from NICE Appendix H Quality appraisal checklist – qualitative studies

***3 How defensible/rigorous is the research design/methodology?***		
Is the design appropriate to the research question?	Defensible	Comments
Is the rationale given for using qualitative approach?	Indefensible	
Are there clear accounts of the rationale/justification of the sampling, data collection, and data analysis techniques used?	Not sure	
***8 Is the analysis sufficiently rigorous?***		
Is the procedure explicit – is it clear how the data was analysed to arrive at the result?	Rigorous	Comments
How systematic is the analysis, is the procedure reliable/dependable?	Not rigorous	
Is it clear how the themes and concepts were derived from the data?	Not sure – not reported	
***Overall assessment***		
As far as can be ascertained from the paper, how well was the study conducted?	++ + -	Comments

### Presentation of findings

Three qualitative studies are included in this integrative review. The first study explores Cypriot midwives’ perceptions of their role as an advocate^19^. The second study explores Australian midwives’ experiences of witnessing a traumatic birth^20^. A third study was included, but with caution. In this third study, only two certified nurse-midwives (CNMs) were included within the study and the majority of the data did not distinguish between the three types of participants (nurses, physicians and CNMs). However, where data were specifically attributed to CNMs, it appears that *Midwifery Abdication* occurred. The study explores the social and environmental conditions that create fluctuating agency for safety in two ‘academic’ birth centres in the USA^21^. More detailed summary is presented in Table 3.

**Table 3:** Summary of papers included

*Authors*	*Topic*	*Methodology design*	*Sample size*	*Location*	*Data collection method*	*Data analysis method*	*Validation of analysis*
Hadjigeorgiou & Coxon^19^	Cypriot midwives’ perception of their role as advocates for normal birth	Qualitative	20	3 public maternity departments Cyprus	Semi structured interview (x10) Observation (x10)	Thematic	Independent researcher
Rice & Warland^20^	Midwives experiences of witnessing traumatic birth	Descriptive qualitative	10 Self-selected snowball	5 different models of care Australia	Semi-structured interview (30-60 minutes)	Thematic	Researchers independently analysed data
Lyndon^21^	Social & environmental conditions creating fluctuating agency for safety in 2 urban academic birth centres	Grounded theory	2 CNM 12 RN 5 Dr	2 teaching hospitals USA 2005-2007	Semi structured interview (x10) Observation (x10) 1 CNM	Constant comparative method, dimensional and situational analysis	Researchers independently analysed data

#### Knowing but failing to act

This construct was identified in the original *Midwifery Abdication* paper^7^ and included many elements, such as other people in the birthing room, communication, cultural safety and/or entering an environment with raised stress levels. Medical dominance was referred to, yet in a very limited way. In all three papers included in this integrative review, the influence of the hierarchal cultural and social obedience within a technocratic environment were strongly evident. Midwives, in two of the studies^19,20^ associated the medicalisation of childbirth via frequent interventions with feelings of being ‘powerless’ and ‘intimidated’. Internally, midwives disagreed with the technocratic (medicalised) model of intervention. Externally, most midwives stayed silent, witnessing or reluctantly becoming part of the technocratic model. Importantly, hierarchical and imbalanced power were cited by some midwives as reasons for their inaction and loss of professional voice, as is cultural acceptance of the woman ‘belonging’ to a medical professional^19,20^.

The acceptance of the medical presumption that a woman’s body will fail so intervention is necessary, juxtaposes the midwifery philosophy of trusting the woman’s body to birth. Midwives in both studies were forced into a vulnerable position as they tried to navigate the differences between the midwifery and medical philosophy, which may or may not be reinforced by practice policies that are not supportive of a midwife’s professional autonomy and scope of practice. They felt challenged and at times fearful to raise their professional voice. One midwife found her voice^20^ and was ‘dragged’ by a fellow midwife from the birthing room, who re-enforced cultural acceptance by: *‘this is what happens…we all know what he does…they’re his clients and there is nothing you can do about it*…’ .

We acknowledge that if a midwife feels overridden by hierarchical and imbalanced cultural power, then consciously or unconsciously her ability to be an advocate for a woman and/ or woman-centred care may by hindered. The need to belong, to fit in and be connected and accepted is natural^22^. Yet in order to belong, some midwives chose to conform and ignore what was happening. They had the professional knowledge, skills and expertise to know certain interventions might not be warranted, nonetheless they consciously chose to remain silent, and did not voice their professional concerns. Thus, the majority of the midwives in these three studies^19-21^ in fact abdicated their professional accountability and responsibility. We would like to highlight some empirical data that might offer some understanding of why the midwives acted in such a way, although we do not condone their behaviour. Actions by fellow midwives are noted in empirical data where (senior) midwives endeavour to influence a subservient reaction of obedience to a decision or behaviour^23^. This particularly applies to junior midwives, who find it difficult to step outside the imposed hierarchy and hence are obedient even if they do not agree with what they are observing or being drawn to participate in^24^. For the midwives in the three studies of this integrative review, some underpinned the accepted (medical) hierarchical cultural and social obedience relationships within these maternity environments. Midwives who did not conform to the accepted environmental norms appeared to place themselves in the tenuous position of powerlessness and censorship from their peers.

Broadening from the empirical data that highlighted that the concept of Midwifery Abdication existed^8^, and the constructs within the concept identified in the later work by Jefford and Jomeen^7^, the concept is confirmed to occur within the wider midwifery literature. However, when analysing the three studies in this integrative review, we believe the constructs of Midwifery Abdication need to be expanded to include: ‘external perceptions of midwifery practice’ and ‘how can reflection facilitate change’.

#### External perceptions of midwifery practice

This new construct links with ‘Internalized Perceptions of Midwifery Practice’ of *Midwifery Abdication*. In other words, a midwife may well internalise what she perceives to be ‘good’ midwifery. However, this new construct highlights that midwives adapt their own social identity within midwifery to align themselves with the external perceptions of ‘good’ midwifery in order to find their place within such a midwifery environment.

Recognition of midwifery as a profession was present in two of the studies^19,20^. Midwives believe and work in the space where childbirth is a natural healthy phenomenon and they trust and value the woman and her body to undertake this event^25,26^. To work collaboratively with women within this philosophy, midwifery education has to support midwives to work autonomously within their full scope of practice^10^. Yet there are some midwifery education programs that appear to be grounded within the medical philosophy or, as Davis-Floyd calls it, the ‘technocratic’ childbirth model^27^. The technocratic (medicalised) model’s philosophy does not believe or trust a woman’s body to birth, rather the presumption is that her body will fail so intervention to limit this risk is a necessity^28^. Within such a learning environment, if the predominant philosophy does not embody the midwifery philosophy, then this has the potential to play a significant role in how a midwife perceives her professional autonomy and scope of practice.

Being an accountable practitioner is incumbent with being an autonomous practitioner. A midwife is the guardian of normal^29^, yet needs to have the knowledge and skills to assess risk, so that appropriate consultation and referral can occur. In a reciprocal partnership relationship, the midwife acknowledges and respects the woman as the final decisionmaker irrespective of perception of risk^30-33^. The technocratic model is the opposite, whereby risk and control are symbiotic and belong with the medical hierarchy^27^. This constructed hierarchical ‘obedience’ culture results in a need to find one’s ‘accepted’ place within such an environment. This is evidenced when all but two Cypriot midwives became silent, feeling unprepared and/or disempowered to take on their professional midwifery role, including acting as an advocate for the woman^19^. We offer a tentative supposition, that even if a midwife is trained within a medical model, such as the Cypriot midwives were, she/he may feel unable to advocate for the woman, because she/he has, either consciously or unconsciously, aligned her/his internal perceptions of midwifery to those of external perceptions. It is worth noting, however, this can also happen within a training program whose focus is supporting childbirth as a normal physiological event that can occur in a home, birth centre or hospital. We suggest that despite one’s internal perceptions of midwifery, if the external environment has a risk philosophy that may or may not be reinforced by practice policies that are not supportive of a midwife’s professional autonomy and scope of practice, and therefore does not support or erodes normality, a midwife may or may not adapt to fit in. Although we acknowledge the sample sizes are small, to support this claim we draw on the types of settings some of the midwives, within these three studies, were employed: a community practice setting and midwifery group practice, and birth centres^20,21^. In other words, the insidious risk philosophy of the technocratic (medicalised) model of intervention and the censorship of fellow midwives promotes obedience^24^, irrespective of the setting.

In the three studies examined in this review, the level of competency and confidence to practice as a midwife were perceived to influence two midwives perception of collegial respect and medical deference to a senior midwife. This may interrelate with the social hierarchy and/or seniority that can influence a subservient reaction of obedience to a decision or behaviour, as noted above. Nevertheless, a lack of midwives’ demographic data in the study, unfortunately, prevents further analysis of this aspect.

It could be postulated, that the lack of professional recognition, as noted by the Cypriot midwives, by fellow health practitioners and ultimately the women, was inevitable as they were educated within the technocratic discourse of childbirth and therefore were more likely to practise in such a way. Further, within the Cypriot Ministry of Health, Nursing and Midwifery Council regulatory framework, midwifery is subsumed under nursing. The International Confederation of Midwives, which represents midwifery associations in approximately 100 countries, provides a definition of a midwife^10^, essential competencies for midwifery practice^34^ and global standards for midwifery regulation^35^. Inherent within these key documents is the tenet that midwives work autonomously within the full scope of practice across the spectrum of childbearing. The World Health Organisation Europe Midwifery Curriculum^36^, which includes Cyprus, embraces these key documents. Nevertheless, the Cypriot midwives interviewed appear to indicate that these key principles did not transcend into the Cyprus education programs.

The differences between two philosophies, technocratic and midwifery, are noted by the Australian midwives in the Rice et al.^20^ study. Whilst trying to stay true to honouring the woman’s expectation and her birth experience and their midwifery philosophy, some midwives found themselves in conflict with the practice they observed or felt forced to participate in. The American CNMs in Landon’s study^21^ speak about traditional hierarchical structure of medicine and distribution of power. In this structure, credence is given to those who can cite the latest research. However, it is unclear from the CNM’s data if this led to a more technocratic environment or not. We acknowledge the focus of the Cypriot midwives’ vulnerability is centred on flaws in midwifery undergraduate and post-graduate education. Some Cypriot midwives cited lack of clinical placement and over-exposure to technocratic environments that limited their ability to embrace a woman-centred philosophy and practise within the full scope of a midwife. Although not related to education, Australian midwives from the Rice et al.^20^ study also felt unable to maintain their full scope of practice and woman-centred philosophy.

#### How can reflection facilitate change

This construct is a consequence of Midwifery Abdication rather than a contributing variable.

Within our self is the need to ensure our actions, or inactions, align with ethical principles of non-maleficence and beneficence^37^. To act in such a way, one must have a moral compass of right and wrong. Intrinsically interwoven in this are feelings of ‘regret’, ‘guilt’ a ‘sense of failure’ and a ‘sense of responsibility’ that can arise if we believe harm has been caused to someone through our actions or failure to act.

None of these feelings is acknowledged by the American CNMs^21^. One Cypriot midwife mentioned feeling ‘bad…guilty’ due to her inaction during an induction of labour. Otherwise these feelings appear implicit. Interestingly, bar this one midwife, the other Cypriot midwives appear to fail to take responsibility for their actions or inaction, rather attributing influences outside their control^19^.

Multiple expressions of ‘regret’, ‘guilt’, ‘sense of failure’ and ‘sense of responsibility’ are explicit in the Rice et al.^20^ study. Further, some midwives expressed feeling traumatised on behalf of the woman when forced to stay silent, be a witness or a participant in the technocratic model of care provided to women they were caring for. In such situations, the midwife did not feel able to prioritise the women’s needs, which further fuelled their feelings of guilt.

#### Prioritisation of the women’s needs

This construct is part of *Midwifery Abdication*. It refers to when a midwife sets out to achieve a woman’s wishes for her ideal birth, ignoring anything or anyone, internal or external to the environment, that might derail it^7^. Drawing on the implicit data noted in the three studies of this integrative review, midwives practice does not always facilitate prioritisation of the women’s needs. Rather, on the whole, the midwives were placed in a vulnerable position trying to balance the women’s wishes with situational factors such as: technocratic environment, risk philosophy, and a need to conform. The findings do not appear to be dependent on traditional maternity settings as midwives in the Rice et al.^20^ study were recruited from a community practice setting and midwifery group practice, whilst the two CNMs were from birth centres^21^.

## DISCUSSION

We contend that from the findings noted in the three studies, it would appear that *Midwifery Abdication* can be validated as a potentially global construct, yet acknowledge such a claim is limited by the fact that only three countries were represented in this integrative review. Further, irrespective of the level to which a philosophy of midwifery is adopted, challenges remain for midwives, as demonstrated for these three countries. Importantly, the findings of this review have led to an extension of the constructs of *Midwifery Abdication*. ‘External Perceptions of Midwifery Practice’ is a new construct and is where external perceptions of midwifery practice and internal perceptions of what constitutes ‘good’ midwifery practice are intrinsically linked and seemingly influential in creating a midwife’s professional identity. Notably, the degree at which the midwifery philosophy is implicitly and/or explicitly coupled to the extent of the technocratic philosophy in any given environment appears to play a significant role. Another substantial influencing factor that may result in a midwife abdicating her role is the constructed hierarchy ‘obedience’ culture and the associated need to find an ‘accepted’ place within such an environment.

Such environments seemingly impact, at differing but significant degrees, on a midwife’s ability to fully exercise autonomy, and to advocate for women and normality of birth. We suggest that if the environment has a risk philosophy, which may or may not be reinforced by practice policies that support a midwife’s professional midwifery autonomy and scope of practice, it may lead to the erosion of midwifery; hence creating a context where *Midwifery Abdication* is inevitable or at least very difficult to prevent. We would argue, however, that when midwives find themselves in such a contested space, governed by technocratic assumptions and practices irrespective of setting, they should embrace their professional status as autonomous practitioners. We acknowledge this is highly challenging for midwives but failure to do so risks abdication of professional accountability and responsibility to the contextual (technocratic) environment, resulting inevitably in Midwifery Abdication. Midwifery education programs, both undergraduate and postgraduate, as noted in the Cypriot education program, can play a significant role in supporting (student) midwives to become empowered practitioners. In addition, the continued generation of evidence related to the benefits of woman-centred care and the positive impact of an effective midwife-woman relationship on women’s satisfaction and psychological health outcomes, is critical as a counteraction to the risk-focused evidence base.

## CONCLUSIONS

Where midwives consciously abdicate their professional role and fail to prioritise the woman’s needs over someone or something within or external to the environment, as noted above, they experience feelings of regret and guilt. Reflection appears to be a key element noted in such situations and some midwives, within the three studies included in this integrative review, appear to engage in reflection whilst others do not. When the reflection becomes reflexive, it appears to have personal and professional consequences, but it only facilitates change for both midwives and women, if those midwives who reflect do change their behaviours. It could be postulated that a reason some midwives do not engage in reflection and reflectivity is that it becomes too challenging and even potentially traumatic. As a result, this impacts further upon current and future interactions and a vicious cycle is created. If midwives were given the support to reflect safely and meaningfully and to develop leadership skills, this may allow them to feel enabled and in a position of power, so they could challenge dominant norms and lead practice and culture changes. We acknowledge that whilst this topic may be challenging, it would seem an area worthy of further debate and empirical investigation.

## CONFLICTS OF INTEREST

Authors have completed and submitted the ICMJE Form for Disclosure of Potential Conflicts of Interest and none was reported.
